# Improvement of Menopausal Symptoms by *Beta vulgaris*, *Artemisia princeps*, and *Eleutherococcus senticosus* via Estrogen Pathway Activation in MCF‐7 Cells and OVX Mice

**DOI:** 10.1002/fsn3.71211

**Published:** 2025-11-14

**Authors:** Tae‐baek Lee, Eunju Jang, Soobin Choi, Lisa Tonini, Da‐Ye Nam, Jae‐Hoon Kim, Hyuk‐Joon Choi, Changhwan Ahn

**Affiliations:** ^1^ Laboratory of Veterinary Physiology, College of Veterinary Medicine Jeju National University Jeju Republic of Korea; ^2^ College of Veterinary Medicine, Veterinary Medical Research Institute Jeju National University Jeju Korea; ^3^ BK Bio Jeju Republic of Korea

**Keywords:** estrogen signaling pathway, menopause symptom management, plant‐based candidates, plant‐derived phytoestrogen

## Abstract

Menopause, a natural transition marked by estrogen decline, is a growing public health concern because it impairs quality of life. Hormone replacement therapy (HRT) has been widely employed to alleviate menopausal symptoms, including osteoporosis, uterine atrophy, vaginal dryness, fat redistribution, and increased cardiovascular risk, but use is limited by adverse effects including hormonal imbalance and increased cancer risk. As potential alternatives, plant‐derived phytoestrogens are actively investigated, yet validated candidate extracts remain scarce. Here we evaluated the estrogenic activity of three extracts— *Beta vulgaris* , *Artemisia princeps*, and *Eleutherococcus senticosus* —using ER‐positive MCF‐7 cells and an ovariectomized (OVX) mouse model. From an initial screen, three extracts induced estrogen‐mediated proliferation in ER‐positive MCF‐7 cells. In OVX mice, efficacy varied by phenotype: *B. vulgaris* and *A. princeps* produced more pronounced improvements, whereas *E. senticosus* was less effective on several endpoints. Mechanistically, *B. vulgaris* and *A. princeps* engaged both genomic and non‐genomic estrogen signaling, while *E. senticosus* showed inconsistent activation, suggesting a more limited or alternative mode of action. Overall, efficacy varied across extracts: *E. senticosus* had modest activity, whereas *B. vulgaris* and *A. princeps* produced robust, reproducible improvements, making them promising for plant‐based management of menopausal symptoms.

## Graphical abstract

Plant extracts from *Beta vulgaris* (BK‐R) and *Artemisia princeps* (BK‐A) act as full estrogen‐mimetics, activating both ERα genomic and non‐genomic signaling in MCF‐7 cells and OVX mice. Across in vivo assays, BK‐R/BK‐A restored uterine structure, improved estrous cycling, preserved trabecular bone, and normalized serum E2/osteocalcin, approximating estradiol benzoate. *Eleutherococcus senticosus* (BK‐E) showed weaker, primarily genomic activity, suggesting tissue‐selective modulation and positioning BK‐R/BK‐A as promising phytoestrogen candidates for menopausal symptom management.

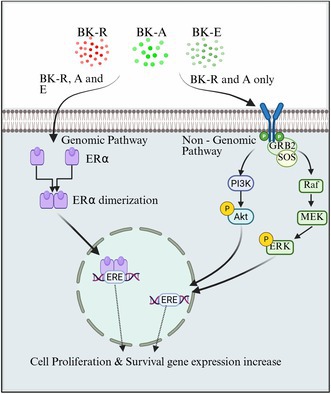

## 1 Introduction

Menopause is the natural cessation of ovarian function, resulting in decreased estrogen levels leading to osteoporosis, metabolic disorders, uterine and vaginal atrophy, and cardiovascular risks, affecting women's health and quality of life (Crandall et al. 2023; Davis et al. 2015; El Khoudary et al. 2019; Manson et al. 2024; Mou et al. 2025).

Although hormone replacement therapy (HRT) has been the standard treatment for menopausal symptoms, there are still concerns regarding its association with gynecological cancers and metabolic dysregulation (Chlebowski et al. 2020; Collaborative Group on Hormonal Factors in Breast 2019). Hence, menopausal women choose to seek alternative plant‐based therapies (Borrelli and Ernst 2010; Johnson et al. 2019), which exhibit natural origin safety profiles (Atanasov et al. 2021). Previous researchers have already illustrated that ginkgo ( *Ginkgo biloba* ), cannabis ( *Cannabis sativa* ), and artemisinin from *Artemisia annua* can be used to effectively treat neurological, infectious, and metabolic diseases (Nelson 2004; Newman and Cragg 2016; Tintore et al. 2019).

Among plant‐derived compounds, phytoestrogens have emerged as promising alternative candidates, as they share structural and functional similarities to estrogen (Blaszczuk et al. 2022; Karalis et al. 2023; Kutuk and Kaplan 2025). This ability to modulate estrogenic activity with fewer side effects than synthetic HRT has positioned them as attractive options in the development of safer, natural treatments for hormone‐related conditions (Glisic et al. 2018; Peng et al. 2019; Wolters et al. 2020).

In this study, we investigated the estrogenic activity of five medicinal plants— *Beta vulgaris* , *Artemisia princeps*, *Eleutherococcus senticosus* , *Centella asiatica* , and *Paeonia lactiflora* —which have been traditionally used in various systems of medicine (Ansari et al. 2025; Mazzio and Soliman 2009). These plants are commonly associated with anti‐inflammatory and antioxidant properties and have also been studied for their potential to influence hormonal balance, immune responses, and metabolic regulation (Banerjee et al. 2023; Brinkhaus et al. 2000; Chung 2017; Clifford et al. 2015; de Oliveira et al. 2021; He and Dai 2011; Siwan et al. 2022; Sun et al. 2023; Taleghani et al. 2020; Zhang and Wei 2020).

Although preliminary evidence suggests that some of these plants may influence hormonal activity, the estrogenic effects of their crude extracts have not been sufficiently examined.

Accordingly, we investigated the estrogenic effects of the crude plant extracts through functional assays in both in vitro and in vivo settings. ER‐positive MCF‐7 cells and ovariectomized (OVX) mice were used as representative models for estrogen‐deficient conditions (Bodinet and Freudenstein 2004; Luengo‐Mateos et al. 2024). Specifically, we categorized the signaling mechanisms into three functional groups based on their mode of action: genomic, non‐genomic, and dual pathways (Azuma and Inoue 2012). Genomic signaling, mediated by ERα‐dependent transcriptional activity, was represented by TFF1, ESR1, PGR, GREB1, and EGR1 (Casimiro et al. 2013; Heldring et al. 2024; Ikeda et al. 2015; Jacquemetton et al. 2021; Liang et al. 2014). Non‐genomic signaling, associated with rapid kinase cascades, was assessed through markers such as BCL2, AKT1, MAPK1, and IL1B (Bratton et al. 2010; Campbell et al. 2001; Jacquemetton et al. 2021; Menazza and Murphy 2016). Genes influenced by both pathways—JUN, CREB1, MYC, and TGFB1—were classified under the dual category (Berto et al. 2018; Janus et al. 2024; Marino et al. 2006; Vrtacnik et al. 2014; Wang et al. 2011). This classification provided a structured basis for a more precise assessment of pathway‐specific estrogenic responses, supporting clearer interpretation of their associated biological effects. In parallel, estrogenic activity at the tissue level was evaluated through histological and morphometric analyses of estrogen‐responsive organs. In the femur, representative skeletal phenotypes, such as trabecular and cortical bone microarchitecture, bone volume fraction, and cortical porosity, were quantified (Chen et al. 2023). In the uterus, endometrial proliferation, lumen area, epithelial thickness, gland number, and overall circularity were measured as indicators of estrogen‐mediated uterine remodeling (Massri et al. 2023; Xu et al. 2016). In the vaginal epithelium, changes in epithelial cell composition and the occurrence of keratinization were assessed as hallmarks of estrogen‐dependent epithelial differentiation (Cora et al. 2015).

This integrated approach allowed us to assess the extracts' potential to restore estrogen‐like signaling. Our findings provide a scientific basis for their application in managing menopause‐related symptoms.

## 2 Materials and Methods

### 2.1 Material and Extraction

Natural extracts were prepared by washing and grinding the raw materials, followed by extraction with 50% ethanol at 55°C for 4 h. The extract was then filtered, and the solvent was removed via rotary evaporation. The plant materials were sourced from a contract farm located in Gujwa‐eup, Jeju, Republic of Korea, under an exclusive supply agreement with BK Bio (Jeju, Korea). All plant materials were processed into powdered extracts by BK Bio following standardized extraction and drying protocols.

The following standardized extracts were obtained from BK Bio (Jeju, Korea): BK‐R, red beet ( *B. vulgaris* ; Menopause formulation); BK‐A (*A. princeps*; Menopause formulation); BK‐E ( *E. senticosus* ; Menopause formulation); BK‐C ( *C. asiatica* ; Menopause formulation); and BK‐P ( *P. lactiflora* ; Menopause formulation).

### 2.2 Quantification of Chlorogenic Acid Was Carried Out Using High‐Performance Liquid Chromatography (HPLC)

Two systems were employed: the Nexera Lite (Shimadzu, Japan) for the BK‐RedMeno sample, and the UltiMate 3000 (Thermo Fisher Scientific, USA) for the BK‐ArteMeno and BK‐EleMeno samples. For BK‐R, analysis was performed using a Shim‐pack GIS C18 column (4.6 mm × 250 mm, 5 μm) maintained at 40°C, with a PDA detector set at 323 nm. The injection volume was 10 μL, and the flow rate was 1.0 mL/min. The mobile phases consisted of 0.1% trifluoroacetic acid in distilled water (A) and acetonitrile (B). For BK‐A and BK‐E, a Gemini C18 column (4.6 mm × 250 mm, 5 μm) was used under the same temperature and flow rate conditions. Detection was performed using a UV–Vis detector, with an injection volume of 20 μL. The mobile phases were 0.1% formic acid in distilled water (A) and methanol (B).

### 2.3 Cell Culture

The ER‐positive human breast cancer cell line MCF‐7 was obtained from the Korean Cell Line Bank (Seoul, Korea) and cultured in RPMI‐1640 medium (Corning, Corning, NY, USA) supplemented with 10% fetal bovine serum (FBS) (Corning, Corning, NY, USA) and 100 U/mL penicillin–streptomycin (Gibco, Thermo Fisher Scientific, Waltham, MA). The cells were maintained at 37°C in a humidified atmosphere of 95% air and 5% CO_2_. The culture medium was refreshed two to three times per week, and subculturing was performed every 2–3 days at a 1:3 ratio before reaching confluence.

### 2.4 MCF‐7 Cell Viability Assay

To assess the cellular toxicity of extracts, MCF‐7 cells were seeded into 96‐well plates at a density of 5 × 10^3^ cells/well in complete growth medium. After 24 h, the medium was replaced with estrogen‐free medium consisting of phenol‐red‐free RPMI‐1640 (Corning, Corning, NY, USA) supplemented with 10% charcoal‐dextran‐stripped human serum (Invitrogen, Thermo Fisher Scientific, Waltham, MA, USA). Cells were treated with various concentrations of the test material for 72 h. In selected conditions, cells were also co‐treated with the estrogen receptor antagonist tamoxifen (Sigma‐Aldrich, St. Louis, MO, USA). 17β‐Estradiol (Sigma‐Aldrich, St. Louis, MO, USA) and PBS were used as positive and negative controls, respectively.

### 2.5 MCF‐7 Estrogenic Cell Proliferation Assay

To assess estrogen‐mediated activity, MCF‐7 cells were seeded into 96‐well plates at a density of 3 × 10^3^ cells/well in complete growth medium. After 24 h, the medium was replaced with estrogen‐free medium consisting of phenol‐red‐free RPMI‐1640 (Corning, Corning, NY, USA) supplemented with 10% charcoal‐dextran‐stripped human serum (Invitrogen, Thermo Fisher Scientific, Waltham, MA, USA). Cells were treated with various concentrations of the test material for 48 h. In selected conditions, cells were also co‐treated with the estrogen receptor antagonist tamoxifen (Sigma‐Aldrich, St. Louis, MO, USA). 17β‐Estradiol (Sigma‐Aldrich, St. Louis, MO, USA) and PBS were used as positive and negative controls, respectively.

Phase‐contrast images were acquired at 10× under identical settings and processed in ImageJ/Fiji (8‐bit). A single preset threshold, calibrated on control and tamoxifen images, was applied unchanged to all groups, and objects were separated with Watershed as needed. Following published descriptions for ER‐positive MCF‐7 cells (Foo et al. 2019; Prashanth Kumar et al. 2015; Razak et al. 2019), cells appearing bright, highly condensed, and shrunken were classified as growth‐arrest morphology–positive. Raters counted cells individually with the Multi‐Point tool (Counter‐1, positive; Counter‐2, normal/negative). Counts were exported and summarized as cells per field, with ambiguous cases resolved by visual comparison to the published examples.

### 2.6 CCK‐8 Proliferation Assay

After treatment and 2–3 days of incubation, cell proliferation was measured using the Cell Counting Kit‐8 (CCK‐8) assay. A total of 10 μL of CCK‐8 reagent was added to each well of the 96‐well microplate. The plate was then incubated in a 5% CO₂ incubator at 37°C for 2 h to allow the reagent to react with metabolically active cells. After incubation, absorbance was measured at 450 nm using a microplate reader. A standard curve was established by plotting the number of cells on the *x* axis and the corresponding absorbance on the *y* axis, which was then used to estimate the level of cell proliferation in each treatment group.

### 2.7 RNA Isolation and cDNA Synthesis

MCF‐7 cells were seeded into 6‐well plates at a density of 3 × 10^5^ cells/cm^2^ in RPMI‐1640 medium (Corning, Corning, NY, USA) and incubated at 37°C in a humidified atmosphere containing 5% CO_2_. After 24 h, the medium was replaced with estrogen‐free medium, with or without the test compounds. After an additional 2 h incubation, cells were washed with PBS and total RNA was extracted using TRIzol reagent (Invitrogen, Thermo Fisher Scientific, Waltham, MA, USA; Cat. No. 15596026), according to the manufacturer's instructions. RNA concentration and purity were determined by measuring absorbance ratios at A260/A280 using a spectrophotometer. A total of 1 μg of RNA was used for complementary DNA (cDNA) synthesis. The reaction was prepared in a final volume of 20 μL, containing 0.2 μL of random primer (Promega, Madison, WI, USA), 2 μL of 0.1 M DTT (Invitrogen, Thermo Fisher Scientific, Waltham, MA, USA), 4 μL of 5× reverse transcription buffer (Invitrogen), 1 μL of M‐MLV reverse transcriptase (Invitrogen), 1 μL of 10 mM dNTP mix (Intron, Seongnam, Gyeonggi, Korea), and DEPC‐treated water (Invitrogen) to adjust the final volume. The mixture was incubated at 37°C for 1 h, followed by an additional incubation at 4°C for 1 h to stabilize the synthesized cDNA. The resulting cDNA was either used immediately for downstream applications such as PCR or qPCR or stored at −20°C until use.

### 2.8 Real‐Time Reverse Transcription PCR (RT‐PCR)

Gene expression analysis of estrogen‐responsive genes was performed using a one‐step real‐time reverse transcription PCR (RT‐PCR) with the PowerUp SYBR Green Master Mix (Invitrogen) on the QuantStudio Real‐Time PCR System (Thermo Fisher Scientific). The PCR reaction included specific primers targeting the genes of interest, and the total reaction volume followed the manufacturer's recommended protocol. The thermal cycling conditions were as follows: reverse transcription at 50°C for 30 min, initial denaturation at 95°C for 5 min, followed by 45 cycles of denaturation at 95°C for 30 s, annealing at 60°C for 30 s, and extension at 72°C for 30 s. A melting curve analysis was performed from 72°C to 95°C to confirm the specificity of amplification. Selected amplified products were also verified by 2% agarose gel electrophoresis. Cycle threshold (*C*_t_) values were defined as the cycle number at which fluorescence exceeded the background signal. Relative gene expression levels were calculated using the $2^{-∆∆C_{t}}$ method, with beta‐actin used as the internal control for normalization.

### 2.9 Animals and Treatment

Seven‐week‐old female 5B6/C5 mice (Experimental Animal Center of Academy of Military) were obtained from Samtako Bio Korea (Osan, Korea) and acclimatized for 7 days prior to the experiment. Bilateral ovariectomy (OVX) was performed under anesthesia with isoflurane by vaporizer (3% for the induction and 1% for the maintenance) and oxygen (0.5–1.0 L/min). In the sham‐operated group, only the periovarian fat was removed. Following surgery, all mice were allowed a seven‐day recovery period before experimental treatments started. Mice were randomly assigned to the following groups (*n* = 7 per group): sham‐operated with corn oil, OVX with corn oil, OVX treated with estradiol benzoate (EB, 1 μg/150 μL) administered subcutaneously every other day, OVX treated with *B. vulgaris* extract (BK‐R; 50 or 100 mg/kg/day), OVX treated with *A. princeps* extract (BK‐A; 50 or 100 mg/kg/day), and OVX treated with *E. senticosus* extract (BK‐E; 50 or 100 mg/kg/day). All plant extracts were administered orally once daily for 28 days. Vehicle (corn oil) was administered in equal volume in the control groups.

During the final 7 days of the treatment period, daily vaginal smears were collected to assess the restoration of keratinized epithelial cells and normalization of estrous cycling patterns. All animals were housed under specific pathogen‐free conditions with controlled temperature (24°C ± 2°C), relative humidity (55% ± 5%), and a 12‐h light/dark cycle. Food and water were provided ad libitum throughout the study. All procedures were reviewed and approved by the Institutional Animal Care and Use Committee (IACUC) of Jeju National University (approval number: 2022‐0055) and carried out in accordance with the guidelines of the Ministry of Food and Drug Safety, Republic of Korea.

### 2.10 Monitoring Estrus Cycle

Throughout the 4‐week treatment period, the estrous cycle of all mice was monitored by daily vaginal epithelial cell smear testing. Vaginal lavage samples were fixed in 95% ethanol for 10 min and stained with methylene blue for another 10 min. Microscopic observation of keratinized vaginal cells was used to identify the estrus phase. To minimize observer bias, five independent evaluators performed a blinded assessment in which images of five randomly selected cell clusters from each sample were provided for phase determination. All images were evaluated in this manner, and the results were quantified and summarized as bar plots to visualize the distribution of estrus phases across groups.

### 2.11 Analysis of Tissue and Serum

After 4 weeks of treatment, animals were sacrificed by decapitation. Blood samples were collected from the retro‐orbital plexus for analysis of serum estradiol (E2) and osteocalcin using enzyme‐linked immunosorbent assay (ELISA). Both horns of the uterus were dissected and weighed. The left horns of the uterus and vagina were stored at −80°C for subsequent molecular analyses, while the right horns were fixed in 4% polyoxymethylene (formaldehyde) for 24 h for histological evaluation. Additionally, the distal femur, including the femoral joint region, was collected to assess bone tissue morphology. These samples were also fixed, embedded in paraffin, and processed for histological analysis. All fixed tissues were embedded in paraffin, sectioned at 2 μm thickness, mounted on glass slides, and stained with hematoxylin and eosin (H&E). Tissue morphology and structural integrity were evaluated by light microscopy, and representative images were captured using a Cytation 7 imaging system (Agilent, CA, USA). Quantitative image analysis, including uterine thickness and joint structure measurements, was performed using ImageJ software (NIH, Bethesda, MD, USA).

### 2.12 Western Blot

Uterine tissues were homogenized using a Bead Beater (Biospec Products, Bartlesville, OK, USA) in lysis buffer (Invitrogen, Waltham, MA, USA) supplemented with a protease inhibitor (Invitrogen, Waltham, MA, USA). The homogenates were sonicated at 100% amplitude for 10 cycles using a sonicator (Leica Microsystems, Wetzlar, Germany), followed by centrifugation at 14,000 rpm for 15 min at 4°C. The supernatants were collected, and protein concentration was determined using the Pierce BCA Protein Assay Kit (Thermo Scientific, Rockford, IL, USA). For Western blotting, 20 μg of protein was mixed with protein sample buffer (Bio‐Rad, Hercules, CA, USA) and 1× mercaptoethanol (Merrick, Darmstadt, Germany), then subjected to SDS‐PAGE. Proteins were transferred to nitrocellulose membranes (Cytiva, Marlborough, MA, USA) and blocked with 5% BSA in TBS‐T (Hanlab, Seoul, Republic of Korea). Membranes were incubated overnight at 4°C with the following primary antibodies diluted 1:1000 in blocking buffer: mouse anti‐ERα (F‐10) (Santa Cruz Biotechnology, Dallas, TX, USA), rabbit anti‐phospho‐AKT (Ser473) (#9271; Cell Signaling Technology, Danvers, MA, USA), rabbit anti‐AKT (#9272; Cell Signaling Technology), rabbit anti‐phospho‐ERK1/2 (Thr202/Tyr204) (#9101; Cell Signaling Technology), and rabbit anti‐ERK1/2 (#9102; Cell Signaling Technology). Mouse anti‐β‐actin (#3700; Cell Signaling Technology) was used at a dilution of 1:10,000. HRP‐conjugated secondary antibodies (Cell Signaling Technology) were used at 1:2000 for ERα, p‐AKT, AKT, and p‐ERK, and 1:20,000 for β‐actin. Bands were visualized using the ChemiDoc Touch Imaging System (Bio‐Rad, Hercules, CA, USA).

### 2.13 Statistical Analysis

Statistical significance was determined using Duncan's multiple range test for group comparisons when one‐way analysis of variance (ANOVA) indicated significance at *p* < 0.05. For comparisons between two groups, Student's *t*‐test was used.

## 3 Result

### 3.1 Assessment of Extract‐Induced Effects on Estrogen Receptor‐Dependent Growth in MCF‐7

**FIGURE 1:**
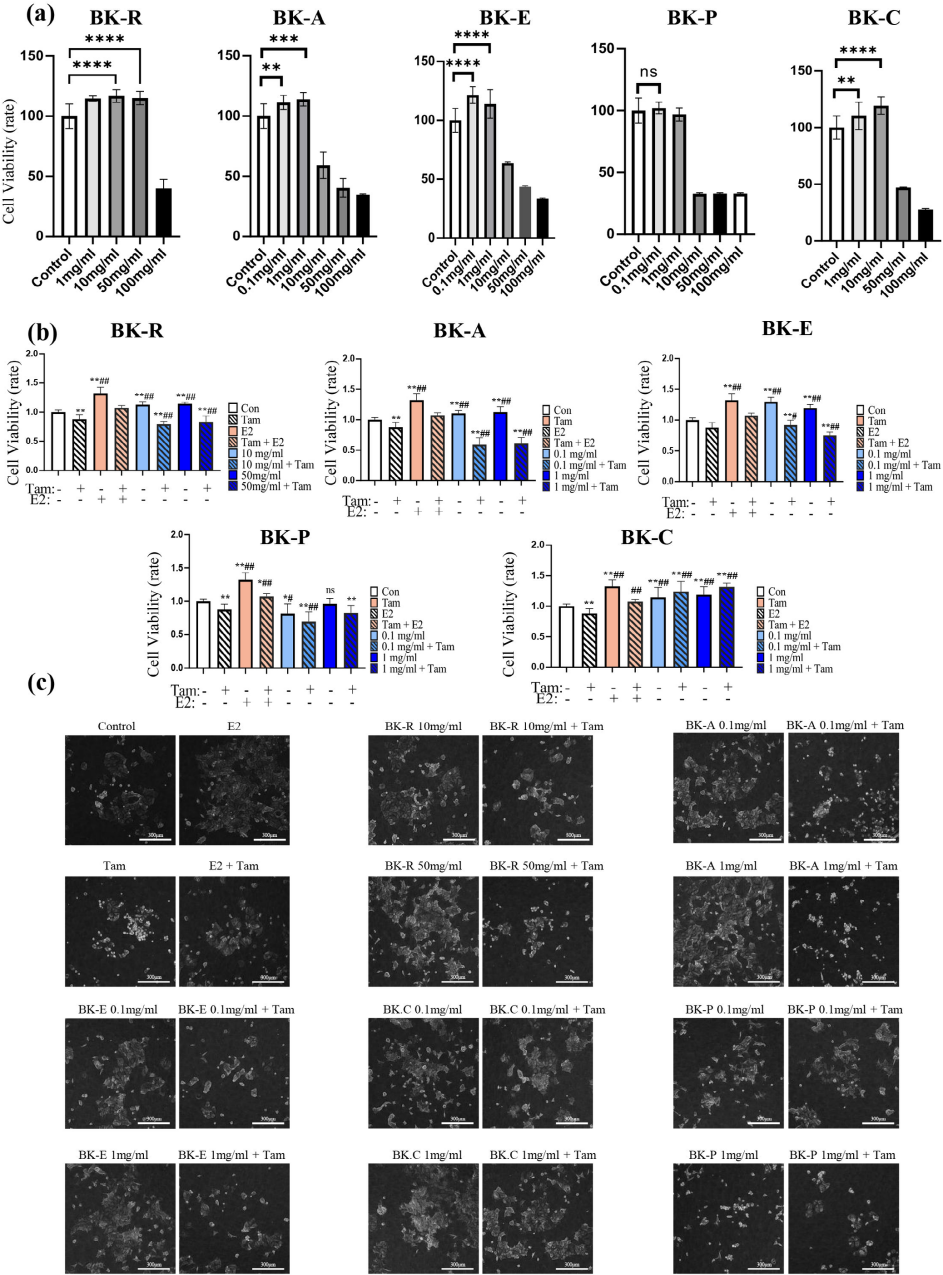
Assessment of extract‐induced effects on estrogen receptor‐dependent growth in MCF‐7 cells. (a) MCF‐7 cells were treated with BK‐R, BK‐A, BK‐E, BK‐P, or BK‐C for 72 h across a broad concentration range. Cell viability (cytotoxicity screen) was measured by CCK‐8. (b) Within the non‐cytotoxic range defined in (a), cells were cultured under estrogen‐deprived conditions and treated for 48 h with each extract ±10 μM tamoxifen. Proliferation was quantified by CCK‐8. E2 (1 nM) and vehicle were used as positive and negative controls. (c) Representative phase‐contrast images (10×) acquired under identical acquisition settings after 48 h in estrogen‐deprived medium. For each extract, images are shown at the same concentrations used in (b), either alone or in combination with 10 μM tamoxifen. Control (vehicle) and E2 (1 nM) are provided as references. Cells with bright, shrunken cytoplasm and condensed appearance illustrate growth‐arrest morphology, whereas polygonal, confluent monolayers indicate proliferation. Only uniform, linear brightness/contrast adjustments were applied to all groups. Same magnification; scale bar = 300 μm. (Quantitative morphology scoring is provided in Figure S1.) Data are presented as mean ± SD (*n* = 12 per group). *p < 0.05, **p < 0.01 vs. blank control group; ^#^ *p* < 0.05, ^##^ *p* < 0.01 vs. tamoxifen group (negative control).

To evaluate the estrogen‐like activity of the extracts on cell proliferation, estradiol (E2) was used as a positive control and tamoxifen, a selective estrogen receptor modulator (SERM), as a negative control. Non‐toxic working concentrations were determined from IC_50_ values obtained in viability assays (Figure 1a). At these concentrations, all extracts—BK‐R ( *B. vulgaris* ), BK‐A (*A. princeps*), BK‐E ( *E. senticosus* ), and BK‐C ( *C. asiatica* )—except BK‐P ( *P. lactiflora* ) significantly increased the proliferation of ER‐positive MCF‐7 cells (Figure 1b).

To determine whether this proliferative effect was ER‐dependent, co‐treatment assays were performed with tamoxifen. In the absence of tamoxifen, BK‐R, BK‐A, and BK‐E did not exhibit cytostatic (growth‐arrest) morphology, consistent with their pro‐growth activity (Foo et al. 2019; Prashanth Kumar et al. 2015; Razak et al. 2019). Upon tamoxifen co‐treatment, growth‐arrest morphology counts became comparable to—or exceeded—those of tamoxifen alone and the growth advantage was lost (Figure 1b,c; Figure S1). By contrast, BK‐C showed no meaningful effect, and BK‐P displayed prominent cytostatic morphology even as a single treatment and failed to increase proliferation. Collectively, these findings indicate that the growth‐promoting actions of BK‐R, BK‐A, and BK‐E are strictly ER‐mediated, as they are abrogated by the SERM tamoxifen.

### 3.2 Analysis of Estrogen Receptor‐Related Signaling in MCF‐7 Cells

**FIGURE 2:**
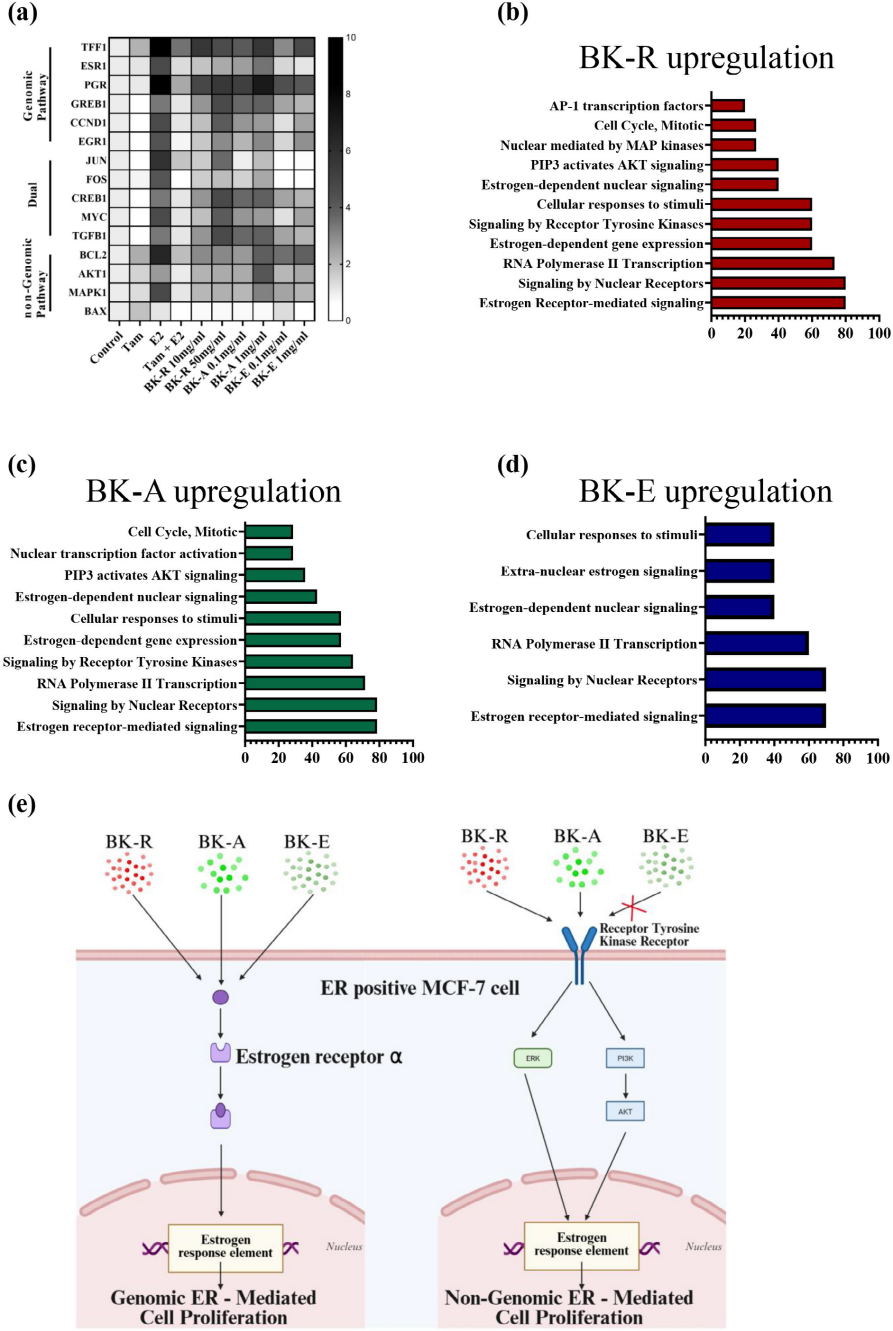
Analysis of estrogen receptor (ER)‐related signaling in MCF‐7 cells treated with BK‐R, A, and E extracts. (a) Heatmap of ER‐related gene expression by RT‐qPCR, normalized to control. Gene Ontology (GO) and Reactome database analysis of upregulated genes in MCF‐7 cells treated with (b) BK‐R, (c) BK‐A, and (d) BK‐E extracts, performed via the DAVID (Database for Annotation, Visualization and Integrated Discovery) bioinformatics tool. (e) Schematic representation of proposed mechanisms by which BK‐R, BK‐A, and BK‐E extracts influence ER signaling pathways. BK‐R and BK‐A activated both ERα‐mediated genomic (nuclear) pathways and genes associated with non‐genomic signaling, whereas BK‐E primarily engaged the genomic arm without detectable activation of non‐genomic pathways. Data are shown as mean ± SD; *n* = 5 independent experiments.

To further investigate the underlying mechanisms, we classified the target genes into three functional categories based on established definitions of estrogen signaling pathways (Figure 2a):

- Genomic pathway, comprising classical estrogen‐responsive genes such as *TFF1*, *ESR1*, *PGR*, *GREB1*, *CCND1*, and *EGR1*.
- Non‐genomic pathway, represented by rapid signaling mediators including *BCL2*, *AKT1*, *MAPK1*, and *BAX*.
- Dual pathway, encompassing transcriptional regulators, such as *JUN*, *FOS*, *CREB1*, *MYC*, and *TGFB1*, which are modulated by both genomic and non‐genomic estrogen signaling.

Gene expression profiles were visualized as a heatmap, and pathway enrichment was analyzed using DAVID (GO and Reactome) to identify extract‐specific functional pathways (Figure 2a–d). Classification of target genes into genomic, non‐genomic, and dual pathways revealed distinct activation patterns for BK‐R, BK‐A, and BK‐E. BK‐R and BK‐A predominantly induced genomic pathways including estrogen receptor‐mediated and nuclear receptor signaling. In parallel, both extracts enhanced receptor tyrosine kinase and PI3K–AKT signaling, indicative of concurrent activation of genomic and non‐genomic cascades (Figure 2b–e). In contrast, BK‐E selectively upregulated extra‐nuclear estrogen signaling and cellular response pathways, consistent with a non‐genomic mechanism independent of AKT/MAPK signaling (Figure 2d). Collectively, these findings demonstrate that while all three extracts engage estrogen‐mediated signaling, BK‐R and BK‐A appear to promote proliferation through dual genomic–non‐genomic activation, whereas BK‐E may exert its effects via an alternative non‐genomic route (Figure 2e).

### 3.3 Schematic Overview and Representative Photomicrographs of Vaginal Smears

**FIGURE 3:**
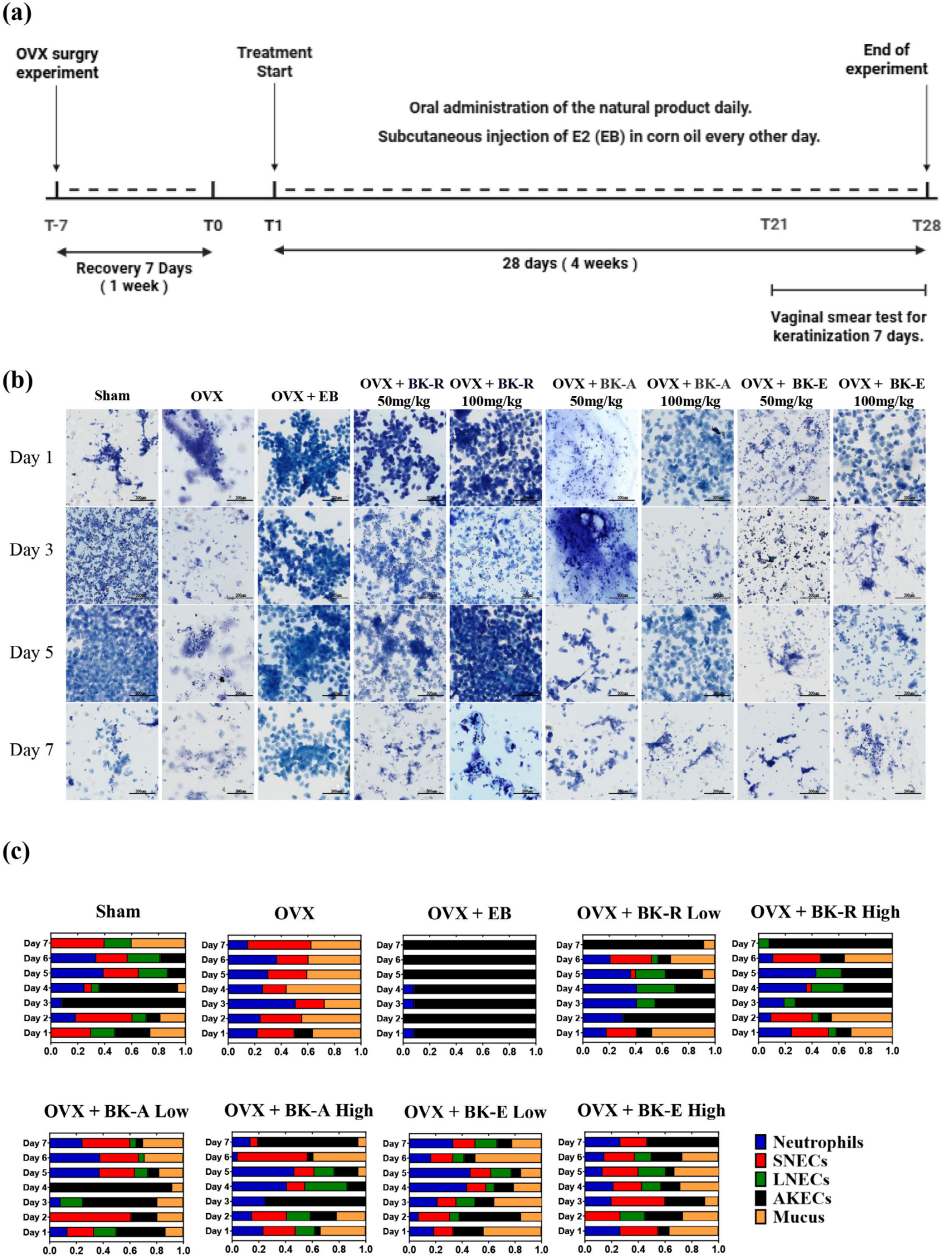
Schematic overview and representative photomicrographs of vaginal smears in OVX mice model. (a) Experimental timeline showing the schedule of ovariectomy (OVX), recovery, daily oral administration of natural products, and subcutaneous injection of estradiol benzoate (EB) every other day. Vaginal smear tests were conducted during the final 7 days to evaluate epithelial cell keratinization and estrous cycle progression. (b) Representative vaginal smear images from Sham, OVX, and OVX + EB groups on Days 1, 3, 5, and 7. The Sham group displayed a normal estrous cycle, while OVX mice showed disrupted cyclicity. EB treatment restored typical estrous cycling patterns, including keratinized epithelial cells. Representative vaginal smear images from OVX mice treated with BK‐R, BK‐A, and BK‐E extracts (50 mg/kg and 100 mg/kg). Treatment with the extracts induced changes in epithelial cell morphology consistent with partial restoration of the estrous cycle, particularly in the BK‐R group, where cycling patterns resembled those of the Sham group. Images are shown at the same magnification (scale bar = 200 μm). (c) Stacked bar graphs showing the proportional distribution of cell types—neutrophils (blue), small nucleated epithelial cells (SNECs, red), large nucleated epithelial cells (LNECs, green), anucleate keratinized epithelial cells (AKECs, black), and mucus (orange)—in vaginal smears collected over 7 consecutive days. The Sham group showed regular cycling, OVX mice exhibited persistent diestrus (SNECs and mucus‐dominant), and EB treatment induced a sustained estrus‐like state rather than restoring normal cyclicity. Extract‐treated groups displayed varying degrees of estrous cycle restoration (*n* = 7 mice per group).

The ovariectomized (OVX) mouse model and vaginal smear testing followed the experimental timeline shown in Figure 3a. To assess the estrogenic activity of the natural extracts BK‐R, BK‐A, and BK‐E, estradiol benzoate (EB), a synthetic estrogen, served as the positive control.

To determine the extent of estrous cycle restoration, we collected vaginal smears from randomly selected mice in each experimental group—Sham, OVX, OVX + E2, and extract‐treated groups (Figure 3a). Vaginal epithelial cell populations were categorized as follows: (1) small nucleated keratinized epithelial cells, (2) large nucleated keratinized epithelial cells, (3) anucleate keratinized epithelial cells, (4) neutrophils (presence/absence and density), and (5) mucus (Cora et al. 2015; McLean et al. 2012).

As shown in Figure 3b, untreated OVX mice remained in a constant diestrus stage, and vaginal smears contained only neutrophils and mucus. These observations confirm complete ablation of ovarian function and the absence of endogenous estrogen. In contrast, EB‐treated OVX mice (OVX + EB) exhibited a predominance of cornified epithelial cells, indicating estrogenic stimulation and maintenance of the estrus phase (Wang et al. 2022).

Notably, oral administration of BK‐R, BK‐A, or BK‐E at 50 or 100 mg/kg partially restored estrous cycling, with mice progressing through proestrus, estrus, metestrus, and diestrus stages during the treatment period (Figure 3b). These cyclic changes suggest that the three natural extracts exert estrogen‐like effects in vivo, potentially promoting uterine hypertrophy and elongation of estrogen‐responsive reproductive tissues.

Vaginal smear cytology over 7 days was used to evaluate keratinization changes and estrous cycle modulation in OVX mice treated with EB or natural extracts (BK‐R, BK‐A, BK‐E). In the Sham group, normal cyclicity was evident, with daily shifts in AKECs, SNECs, mucus, and neutrophils reflecting all estrous stages. OVX mice remained in persistent diestrus, showing minimal AKECs and predominance of mucus, neutrophils, and SNECs. EB treatment produced a sustained estrus‐like state, with AKECs dominant throughout (Cora et al. 2015; McLean et al. 2012; Yeo et al. 2025).

BK‐R low‐dose mice had peak AKECs on Days 2 and 7, persistent neutrophils (> 10% Days 1–6), partial LNEC increases (Days 2–6), and fluctuating SNECs; these changes were more rapid and pronounced in the high‐dose group, with higher overall AKEC levels (Figure 3b,c). BK‐A produced gradual AKEC increases (Days 1–4) followed by a decline to Day 7, daily SNEC and neutrophil changes, and in the high‐dose group, cell composition patterns most closely matched Sham, including mucus trends, suggesting near‐normal cycle restoration without the continuous stimulation seen in EB (Figure 3b,c). BK‐E caused only modest AKEC and neutrophil changes, high mucus levels, minimal SNEC variation, and the smallest cyclic shifts among extracts (Figure 3c).

Overall, BK‐A most effectively normalized estrous cycle patterns, BK‐R induced moderate but faster epithelial turnover, and BK‐E had the weakest effect. Fluctuations in AKECs, SNECs, and mucus proved to be sensitive indicators of estrogenic activity and cycle restoration in OVX mice.

### 3.4 Effects on Physiological and Uterine Changes in OVX Mice Treated

Postmenopausal women exhibit increased blood cholesterol and visceral fat accumulation due to reduced conversion of cholesterol into estrogen, leading to greater abdominal fat deposition (Davis et al. 2015). Consistently, ovariectomized (OVX) animal models gain weight as a result of estrogen deficiency (Xu et al. 2016), which elevates the risk of metabolic syndrome and cardiovascular disease (Everson‐Rose et al. 2021). To assess whether these changes could be mitigated, we monitored total body weight over a 4‐week period in sham‐operated, OVX, OVX + estradiol benzoate (EB), and OVX mice treated with BK‐R, BK‐A, or BK‐E.

**FIGURE 4:**
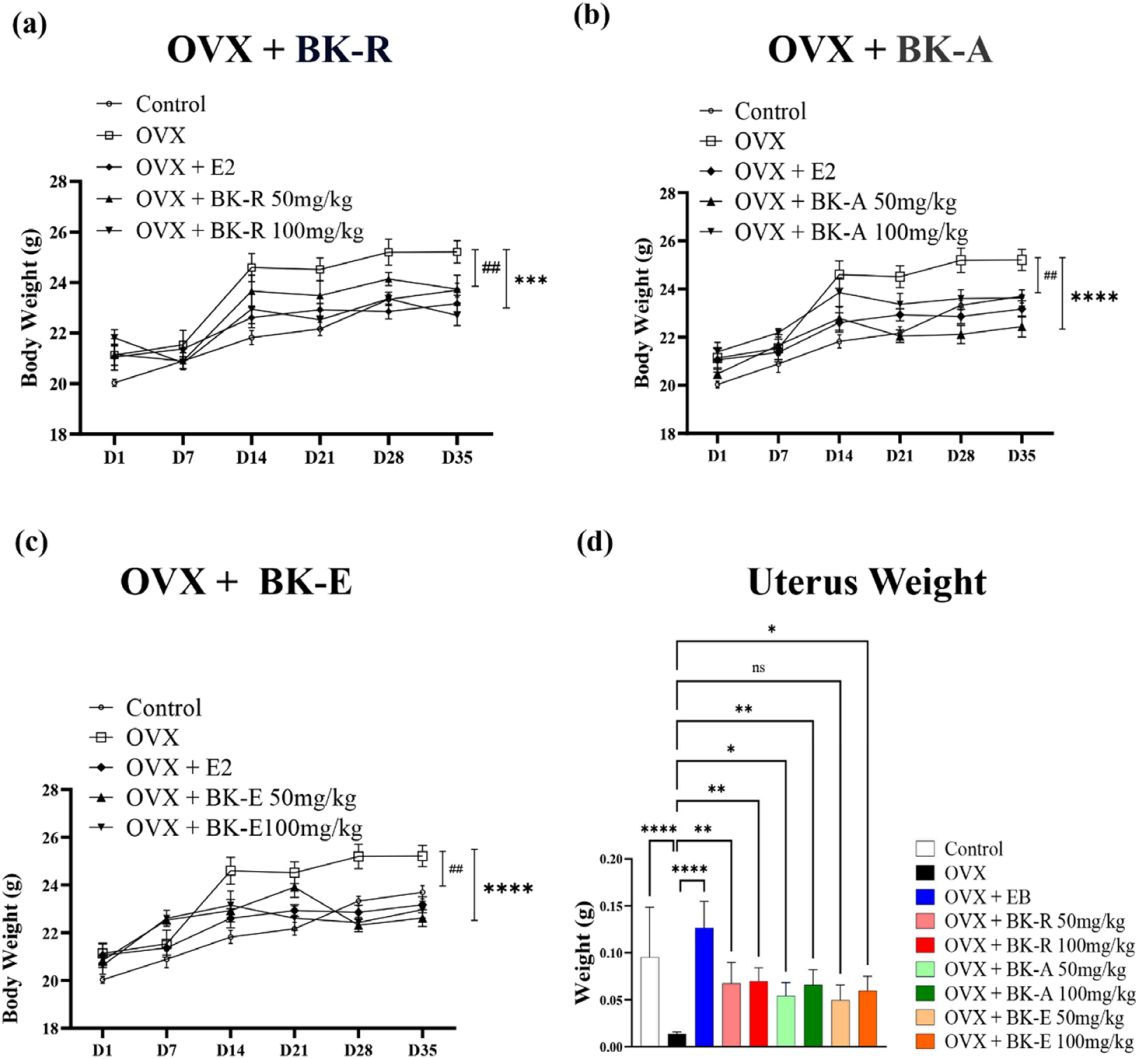
Changes in body weight and uterine tissue among Sham, OVX, and treatment groups in mice. Body weight changes over 35 days in ovariectomized (OVX) mice treated with (a) BK‐R, (b) BK‐A, or (c) BK‐E extracts at doses of 50 and 100 mg/kg. The Sham (control), OVX, and OVX + EB (estradiol benzoate) groups were included for comparison. OVX led to a gradual increase in body weight, which was suppressed by EB and extract treatments, particularly at higher extract doses. (d) Uterine weight measured at the end of the study (Day 35). OVX significantly reduced uterine mass compared to the Sham group. Treatment with EB and extracts partially restored uterine weight, with the strongest effect observed in the OVX + BK‐R 100 mg/kg and OVX + EB groups. Data are expressed as mean ± SD (*n* = 7 per group). Statistical significance: *p < 0.05, **p < 0.01, ***p < 0.001, ****p < 0.0001.

Initial body weights did not differ significantly among groups; however, by the end of the experiment, OVX mice showed a significantly greater weight gain than sham controls. Treatment with EB or any of the three extracts significantly attenuated OVX‐induced weight gain. Body weights in extract‐treated groups remained similar to those in the EB group (Figure 4a–c).

Uterine weight, markedly reduced in OVX mice due to estrogen deficiency, was significantly restored by BK‐R and BK‐A, with 100 mg/kg doses achieving effects comparable to EB. BK‐E did not significantly alter uterine weight (Figure 4d).

These findings indicate that BK‐R and BK‐A exert in vivo estrogenic activity, attenuate OVX‐induced weight gain, and may serve as phytoestrogen candidates for alleviating postmenopausal metabolic and reproductive changes.

### 3.5 BK‐R, BK‐A and BK‐E Antagonized the Histological Atrophy of Uterus

**FIGURE 5:**
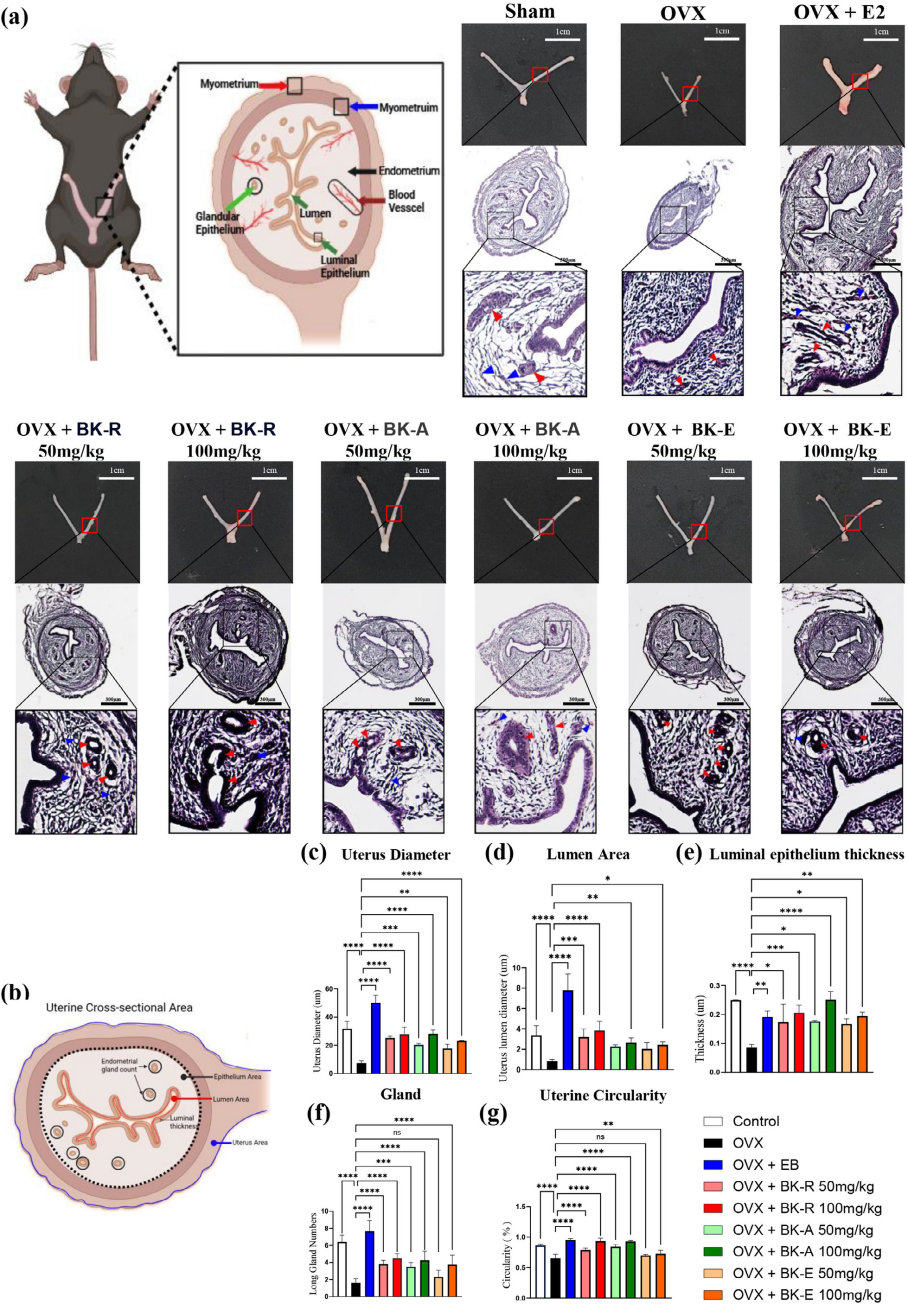
BK‐R, BK‐A and BK‐E antagonized the histological atrophy of uterus in OVX mice. (a) Representative gross and histological images of uteri from Sham, OVX, OVX + EB (estradiol benzoate), and OVX mice treated with BK‐R, BK‐A, or BK‐E (50 or 100 mg/kg). OVX led to uterine atrophy and reduced epithelial and glandular structures. EB and extract treatments restored uterine morphology to varying degrees, with BK‐R 100 mg/kg showing the most pronounced effect. Red arrows indicate the developed gland, and blue arrows indicate blood vessels. Gross uterine images include a 1 cm scale bar, and histological images include a 300 μm scale bar. Histological sections were captured at 10× magnification, with square inset regions further enlarged to an equivalent of 40× magnification. Quantitative analysis of uterine histological parameters: (b) schematic representation of the uterine cross‐sectional area, (c) uterine diameter, (d) lumen area, (e) luminal epithelium thickness, (f) number of endometrial glands, and (g) uterine circularity index (%). Data are expressed as mean ± SD (*n* = 7 per group). Statistical significance: *p < 0.05, **p < 0.01, ***p < 0.001, ****p < 0.0001.

As previously reported, the endometrium of untreated OVX mice consisted of a single layer of columnar epithelial cells, with no detectable mitotic activity (Figure 5a) (Bouxsein et al. 2005). In contrast, all extract‐treated groups displayed clear endometrial stimulation. The positive control, estradiol benzoate (EB), elicited classic estrogenic responses including a thickened, multilayered epithelium with pronounced mitotic activity. Consistent with this, uterine morphology in extract‐treated groups was substantially restored relative to OVX controls.

Based on the histological schematic presented in Figure 5b, we quantitatively evaluated multiple uterine parameters. BK‐R and BK‐A treatments produced robust, dose‐dependent increases in total uterine size (Figure 5c), luminal area (Figure 5d), luminal epithelium depth (Figure 5e), number of glands (Figure 5f), and uterine circularity (Figure 5g), effectively reversing OVX‐induced atrophy. These effects were comparable to those of estradiol benzoate (EB) and were accompanied by a marked increase in vascular structures within the endometrial stroma, indicating estrogen receptor–mediated angiogenesis (Figure 5a). By contrast, BK‐E exhibited the same general trend but with consistently smallest improvements across all parameters, suggesting weaker estrogenic activity relative to BK‐R and BK‐A, without indications of excessive proliferative stimulation.

Taken together, BK‐R and BK‐A restore uterine structure in estrogen‐deficient mice at levels comparable to EB, while BK‐E produces a directionally similar yet attenuated response, without evidence of excessive proliferative stimulation.

### 3.6 Anti‐Osteoporotic Effects in OVX Mice

According to previous studies on the femur of postmenopausal women, the decline in estrogen levels promotes osteoclast formation, accelerating bone resorption and impairing bone remodeling (Eastell et al. 2016; Gossiel et al. 2016). Similarly, OVX mice exhibit typical osteoporotic features, including reduced trabecular thickness and number, along with increased cortical bone porosity, ultimately leading to decreased bone strength (Bouxsein et al. 2005). Based on this background, we investigated whether BK‐R ( *B. vulgaris* ), BK‐A (*A. princeps*), and BK‐E ( *E. senticosus* ) could prevent estrogen deficiency–induced bone loss in the OVX mouse model by assessing femoral microstructural changes.

**FIGURE 6:**
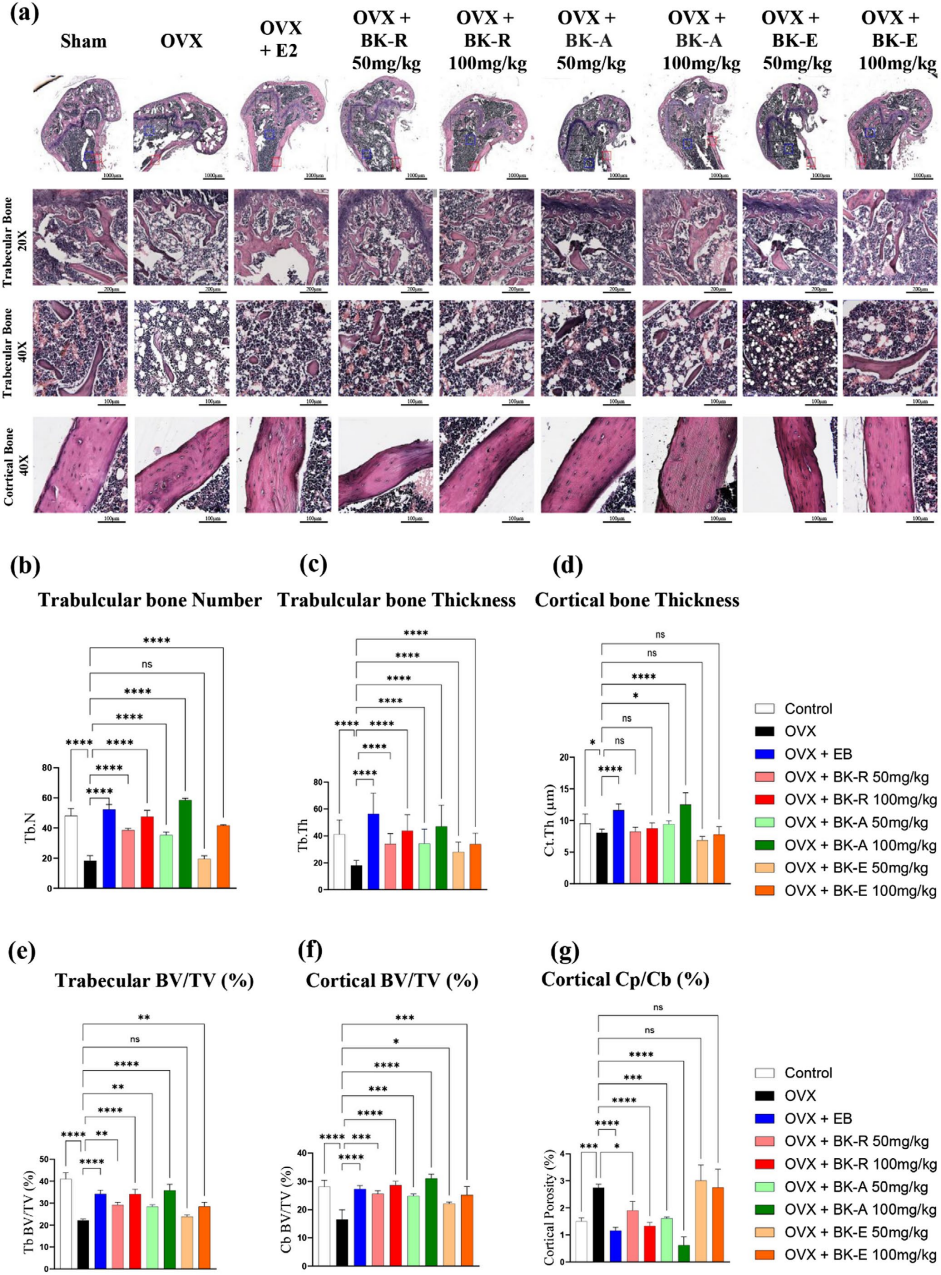
Antiosteoporotic effects of BK extracts in OVX mice. (a) Representative images from Control‐Sham, OVX, and OVX + EB groups, as well as OVX mice treated with BK‐R, BK‐A, or BK‐E extracts at doses of 50 or 100 mg/kg. Each set shows a low‐magnification overview (top row, scale bar = 1000 μm), followed by magnified views of trabecular bone at 20× (scale bar = 200 μm) and 40× (scale bar = 100 μm), and cortical bone at 40× (scale bar = 100 μm). Compared with the OVX group, which exhibited marked trabecular bone loss and cortical thinning, the OVX + EB and BK‐treated groups showed varying degrees of improvement in bone microarchitecture, particularly at higher doses. Quantitative histomorphometric analysis revealed that BK‐R and BK‐A at 100 mg/kg significantly increased (b) trabecular bone number and (c) thickness, enhanced (d) cortical bone thickness, improved both (e) trabecular and (f) cortical bone volume fraction, and reduced (g) cortical porosity, with effects comparable to those observed in the OVX + EB group. Data are presented as mean ± SD (*n* = 7 per group), and statistical significance was determined by one‐way ANOVA followed by post hoc tests (*p < 0.05, **p < 0.01, ***p < 0.001, ****p < 0.0001; ns, not significant).

To assess these effects, we examined structural changes in the femur. OVX mice exhibited increased porosity, cortical thinning, and marked trabecular loss in the distal femur (Figure 6a), confirming successful induction of the estrogen‐deficient model. In contrast, extract‐treated groups showed preserved trabecular architecture with greater density and reduced separation compared with OVX controls, comparable to the EB group (Figure 6a). All extract‐treated groups had significantly higher trabecular number (Tb.N) and thickness (Tb.Th) than OVX mice (Figure 6b,c). Cortical bone thickness (Cb.Th) improved significantly only in the BK‐A group, whereas BK‐R and BK‐E showed no difference from OVX controls (Figure 6d).

To evaluate whether the extracts preserved bone volume and reduced porosity, bone volume fraction (BV/TV) was measured in both trabecular (Tb.BV/TV) and cortical (Ct.BV/TV) compartments, and cortical porosity (Cp/Cb) was assessed. BV/TV values were higher in all extract‐treated groups compared with OVX controls, indicating improved structural preservation (Figure 6e,f). Cortical porosity (Cp/Cb) was markedly elevated in OVX mice but substantially reduced in the BK‐R and BK‐A groups to levels comparable to or lower than those in the EB group, whereas BK‐E showed no improvement (Figure 6g). Collectively, these results indicate that BK‐A and BK‐R substantially mitigate bone loss by preserving trabecular architecture and reducing cortical porosity, whereas BK‐E confers only modest protection and shows the smallest overall effect.

### 3.7 BK‐R and BK‐A Stimulate Both Genomic and Non‐Genomic Pathways via ERα in the Uterus

**FIGURE 7:**
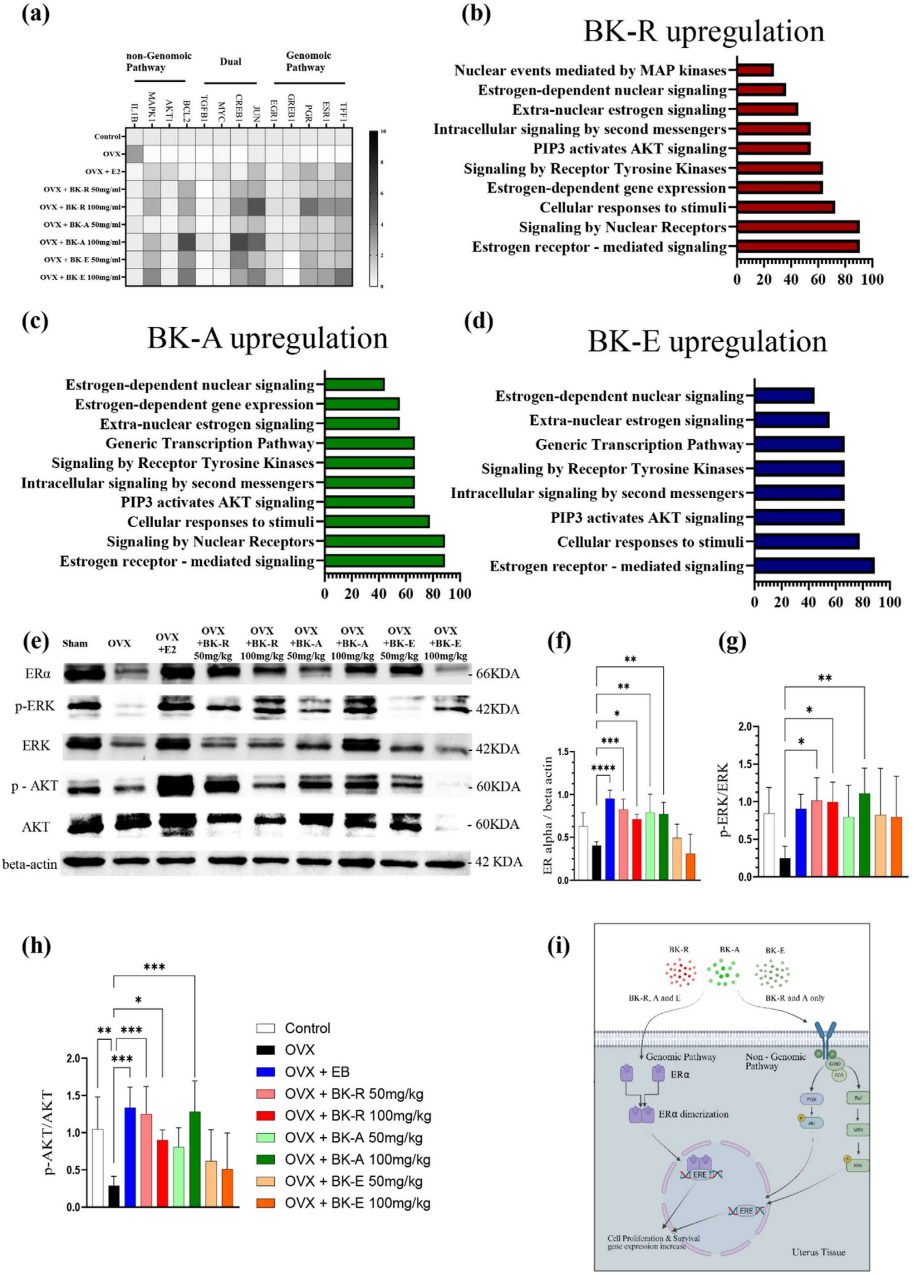
Bk‐R and BK‐A stimulate both genomic and non‐genomic pathways via ERα in the uterus. (a) Heatmap showing expression profiles of genes associated with estrogen receptor signaling in uterine tissues across all groups, categorized into genomic, non‐genomic, and dual pathways, as determined by RT‐qPCR analysis and normalized to control. Functional enrichment analysis of upregulated genes in the uterus following treatment with (b) BK‐R, (c) BK‐A, and (d) BK‐E, performed using the GO (Gene Ontology) and Reactome databases via the DAVID (Database for Annotation, Visualization and Integrated Discovery) bioinformatics tool. (e) Western blot analysis of ERα, p‐ERK, ERK, p‐AKT, AKT, and β‐Actin levels in uterine tissues. Densitometric quantification of (f) ERα normalized to β‐Actin, (g) p‐ERK/ERK ratio, and (h) p‐AKT/AKT ratio indicated activation of both genomic and non‐genomic estrogen signaling pathways in BK‐R and BK‐A groups. (i) Schematic illustration summarizing the proposed mechanisms by which BK‐R and BK‐A activate ERα through both nuclear and extra‐nuclear pathways, and BK‐E activates ERα primarily through nuclear pathways. Data are presented as mean ± SD (*n* = 4 per group). Statistical analysis was performed using one‐way ANOVA followed by post hoc tests (*p < 0.05, **p < 0.01, ***p < 0.001, ****p < 0.0001).

We previously analyzed gene expression in cellular experiments; however, to validate these findings in an OVX model, we further evaluated gene activation in uterine tissue. Gene expression was categorized into three pathways (Figure 7a):

- Genomic pathway, represented by *TFF1*, *ESR1*, *PGR*, *GREB1*, and *EGR1*.
- Non‐genomic pathway, assessed through *BCL2*, *AKT1*, *MAPK1*, and *IL1B*.
- Dual pathway, involving *JUN*, *CREB1*, *MYC*, and *TGFB1*, which are influenced by both genomic and non‐genomic signaling.

Gene expression analysis confirmed that all three extracts (BK‐R, BK‐A, and BK‐E) activated estrogen receptor‐mediated signaling and estrogen‐dependent gene expression. Furthermore, PI3K/AKT and Receptor Tyrosine Kinase (RTK) signaling pathways were also upregulated (Figure 7b–d).

Since the BK‐E results differed from those of previous in vitro experiments, we conducted a more detailed protein‐level analysis. Specifically, we evaluated the activation of ERα, pERK/ERK and pAKT/AKT at the protein level. As a result, increased expression of the ERα, pERK, and pAKT protein ratios was observed only in the BK‐R and BK‐A groups, while BK‐E showed no such elevation at the protein level (Figure 7e–h).

Collectively, the data demonstrate that only BK‐R and BK‐A exhibited estrogen‐like activity in vivo. These extracts activated both genomic (ERα‐dependent transcription) and non‐genomic (PI3K/AKT and MAPK/ERK) signaling pathways, suggesting their action occurs through estrogen receptor‐mediated mechanisms. In contrast, BK‐E showed no significant activation, consistent with weak or negligible engagement of estrogen receptor signaling BK‐E showed no significant activation, consistent with weak or negligible engagement of estrogen receptor signaling (Figure 7e,f).

For clarity, we also note in the text and legend that Figure 7i is a conceptual schematic that summarizes dual‐pathway activation for BK‐R and BK‐A only and does not imply non‐genomic activation for BK‐E; any transcript‐level signals for BK‐E should be interpreted in light of absent ERα, pERK, and pAKT activation at the protein level (Figure 7i).

### 3.8 BK‐R and BK‐A Restore Serum Estrogen and Suppress Osteocalcin Levels in OVX Mice

**FIGURE 8:**
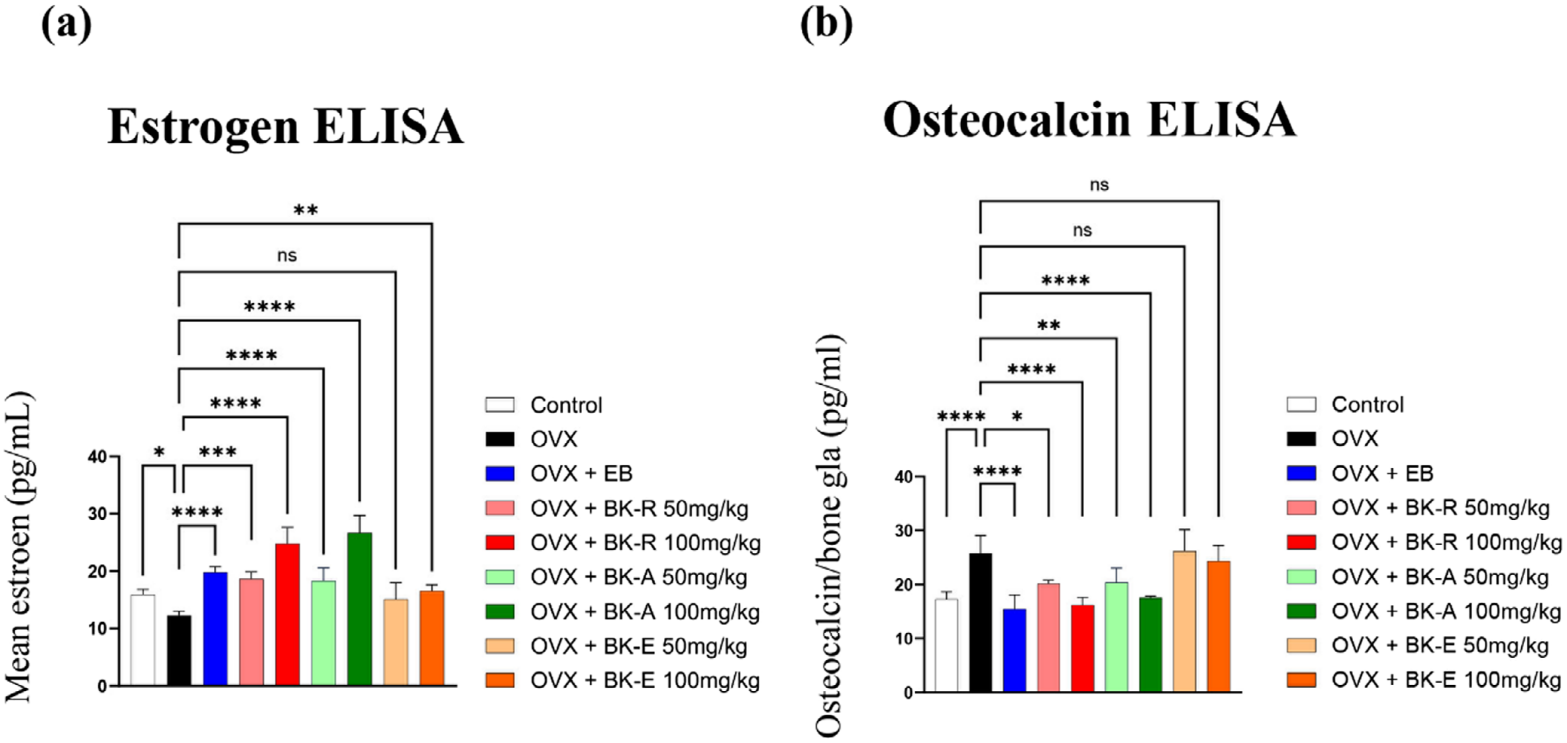
BK‐R and BK‐A restore serum estrogen and suppress osteocalcin levels in OVX mice. (a) Serum estradiol (E2) levels measured by ELISA. Ovariectomy (OVX) significantly reduced serum E2 levels, while treatment with BK‐R and BK‐A (especially at 100 mg/kg) significantly restored E2 to near‐sham or OVX + EB levels. (b) Serum osteocalcin levels, a marker of bone turnover, measured by ELISA, were significantly increased in the OVX group compared to controls. BK‐R and BK‐A administration dose‐dependently reduced osteocalcin levels, suggesting inhibition of excessive bone resorption. Data are presented as mean ± SD (*n* = 7 per group). Statistical significance was determined using one‐way ANOVA followed by post hoc testing (*p < 0.05, **p < 0.01, ***p < 0.001, ****p < 0.0001).

To assess the estrogenic activity of the extracts, serum levels of 17β‐estradiol (E2) and osteocalcin were measured after treatment. OVX mice exhibited a significant reduction in E2 levels compared to the control group, confirming estrogen deficiency. As expected, EB treatment restored serum estradiol (E2) concentrations to levels comparable to those of the control group. Among the extract‐treated groups, only BK‐R, BK‐A, and BK‐E at 100 mg/kg significantly increased serum E2, suggesting their capacity to stimulate endogenous estrogen synthesis or exert estrogen‐mimetic effects. In contrast, BK‐E at 50 mg/kg did not produce a significant change in E2 levels (Figure 8a).

Serum osteocalcin, a biochemical marker of bone formation that is typically elevated under conditions of high bone turnover, was markedly increased in OVX mice. EB treatment normalized osteocalcin levels to those of the control group. Similarly, BK‐R and BK‐A treatments significantly reduced osteocalcin concentrations, restoring them to values comparable to the control and EB groups. BK‐E, however, elicited no significant change in osteocalcin levels (Figure 8b).

Collectively, these findings indicate that BK‐R and BK‐A exert in vivo estrogen‐like effects, both in hormonal regulation and bone metabolism, whereas BK‐E demonstrates limited efficacy in these endpoints.

### 3.9 HPLC‐Based Quantification and Profiling of Major Bioactive Constituents in BK‐R, BK‐A, and BK‐E

**FIGURE 9:**
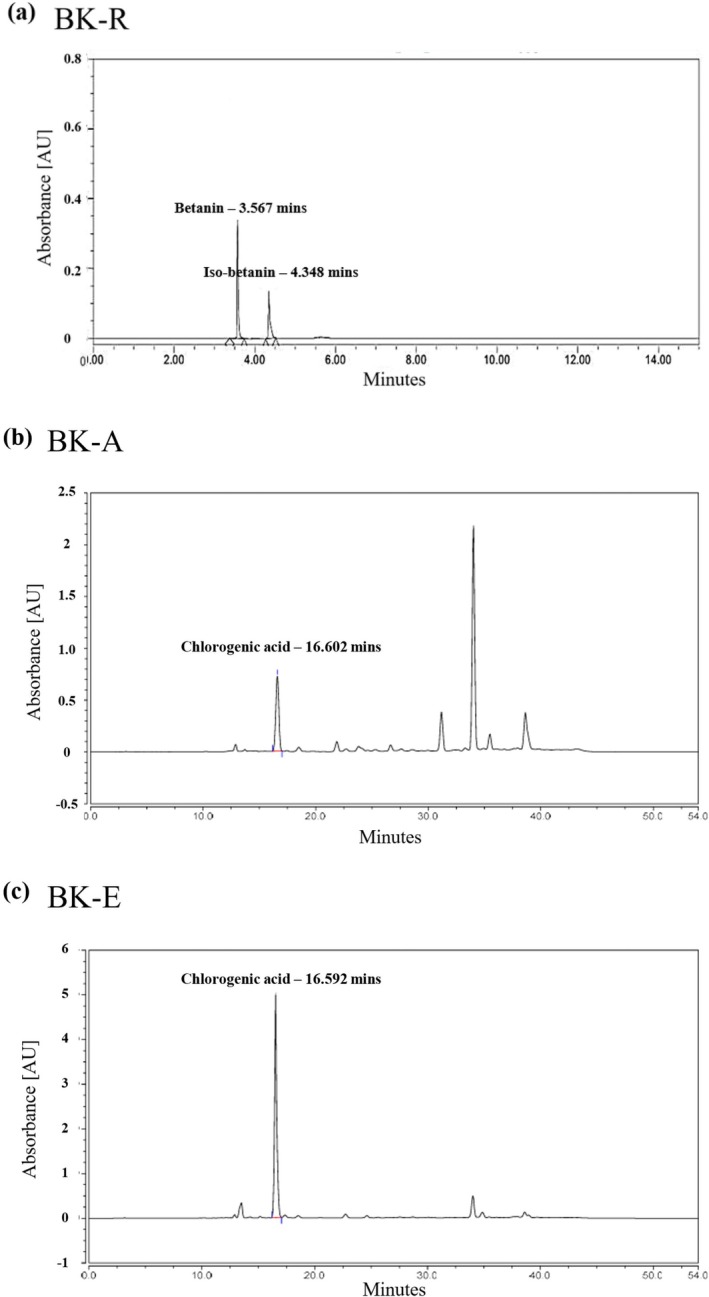
HPLC‐based quantification and profiling of major bioactive constituents in BK‐R, BK‐A, and BK‐E. (a) BK‐R: High‐performance liquid chromatography (HPLC) revealed two predominant peaks corresponding to betanin (retention time = 3.567 min) and iso‐betanin (retention time = 4.348 min), with negligible detection of chlorogenic acid (CGA). These betalain pigments were present in substantially higher relative abundance compared with any other detectable compounds. (b) BK‐A and (c) BK‐E: In contrast, both extracts exhibited CGA as the principal detectable peak (retention time = 16.602 and 16.547 min, respectively), with no significant presence of betanin derivatives. This compositional difference suggests that the pronounced estrogenic activity observed for BK‐R is not attributable to CGA content, but rather may arise from bioactive betanin‐related compounds. Chromatographic separation was monitored by UV absorbance, and compound identities were confirmed by retention time matching with authentic standards under identical chromatographic conditions.

To define the chemical drivers underlying the estrogen‐like activities of BK‐R, BK‐A, and BK‐E, we profiled major constituents by HPLC (Figure 9). BK‐R was enriched in betalain pigments (betanin and iso‐betanin) and contained only minimal chlorogenic acid (CGA), indicating that its strong estrogenic activity is not attributable to CGA (Figure 9a). In contrast, both BK‐A and BK‐E contained CGA as a dominant phenolic, yet their biological effects diverged: BK‐A consistently produced stronger estrogenic responses than BK‐E in vitro and in vivo despite similar CGA prominence. This pattern suggests that additional constituents in BK‐A—likely flavonoids or other phenolics—synergize with CGA to enhance efficacy. BK‐E, although CGA‐rich, displayed weaker uterotrophic effects and limited engagement of canonical non‐genomic signaling, while retaining bone‐protective activity in vivo, a profile compatible with tissue selectivity (SERM‐like).

Collectively, these data indicate that CGA content alone does not predict estrogen‐like activity across extracts. Rather, distinct compositional signatures and intra‐extract synergies appear to shape their biological profiles—betalains in BK‐R, multi‐component synergy (including CGA) in BK‐A, and tissue‐selective, comparatively modest efficacy in BK‐E.

## 4 Discussion

This study initially evaluated five plant‐derived extracts—BK‐R ( *B. vulgaris* ), BK‐A (*A. princeps*), BK‐E ( *E. senticosus* ), BK‐P ( *P. lactiflora* ), and BK‐C ( *C. asiatica* )—selected for their ethnopharmacological relevance and reported bioactivities (Brinkhaus et al. 2000; Chung 2017; Clifford et al. 2015; He and Dai 2011; Sun et al. 2023; Taleghani et al. 2020; Zhou et al. 2023). To screen for estrogen‐like activity, we assessed ER‐positive MCF‐7 breast cancer cell proliferation following treatment with each extract at non‐cytotoxic concentrations. BK‐R, BK‐A, and BK‐E increased proliferation of ER‐positive MCF‐7 cells at non‐cytotoxic concentrations, an effect markedly attenuated by tamoxifen co‐treatment. This suppression was accompanied by growth‐arrest–like morphology, increased cell detachment, and features suggestive of cell cycle arrest, indicating that the proliferative effects were at least partly ER‐dependent.

In the cell‐based assays, BK‐R, BK‐A, and BK‐E each promoted estrogen‐mediated proliferation in ER‐positive MCF‐7 cells, with distinct patterns of ER pathway engagement. BK‐R and BK‐A activated both genomic ERα responses—upregulation of classical ER target genes—and non‐genomic signaling‐associated genes, whereas BK‐E induced genomic activation without stimulating non‐genomic pathways, suggesting a more restricted and potentially tissue‐selective mode of action.

Given these findings, we hypothesized that such differences in ER pathway activation might translate into distinct physiological profiles in vivo. To test this, we employed an ovariectomized (OVX) mouse model, a well‐established system for evaluating estrogenic activity, focusing on whether each extract could alleviate estrogen‐deficiency symptoms (Bouxsein et al. 2005; Couse et al. 1997). Key endpoints included uterine endometrial morphology, vaginal keratinization, and bone microarchitecture—tissues and functions known to be regulated by both genomic and non‐genomic ER signaling (Furuminato et al. 2023; Manolagas et al. 2013; Yu et al. 2022).

In OVX mice, all extracts mitigated hallmark consequences of estrogen loss including uterine atrophy, estrous cycle disruption, and body weight gain. BK‐R and BK‐A promoted robust uterine regeneration and marked restoration of cycling, producing effects comparable to those of estradiol benzoate (Luengo‐Mateos et al. 2024). BK‐E showed weaker effects yet improved uterine morphology and estrous cycling, indicating modest but biologically relevant estrogenic activity.

Bone morphometric analysis further demonstrated that all three extracts mitigated menopause‐related bone loss in OVX mice. BK‐R and BK‐A both preserved trabecular structure and prevented marrow cavity expansion, with BK‐A showing the most pronounced effects including a marked increase in cortical thickness. BK‐E also maintained trabecular integrity but to a lesser extent. Improvements were modest and did not reduce cortical porosity, which aligns with a selective estrogen receptor modulator (SERM)‐like profile (Martinkovich et al. 2014; Shang and Brown 2002). These results indicate that while all extracts support skeletal health under estrogen‐deficient conditions, BK‐A may offer the most robust bone‐protective benefits with positive changes in endometrial architecture, BK‐R provides similarly broad estrogenic effects across both reproductive and skeletal tissues consistent with a full ER agonist profile, and BK‐E exhibits the weakest yet more tissue‐selective profile.

Given the differential physiological outcomes observed among the extracts, we inferred that these effects likely reflect significant alterations analogous to those observed in vitro. Based on the in vitro results—where BK‐R and BK‐A activated both genomic and non‐genomic ER pathways, while BK‐E selectively activated genomic targets—we expected a similar pattern in uterine tissue from OVX mice. However, gene expression analysis revealed that all three extracts, including BK‐E, significantly upregulated both genomic and non‐genomic ER pathway genes, indicating a broader activation profile in vivo than predicted from the cell‐based assays. To further investigate this discrepancy, and considering that such differences might arise from post‐transcriptional or protein‐level regulatory mechanisms (Day and Tuite 1998; Zhao et al. 2018), we analyzed ERα protein expression along with the expression and phosphorylation status of the key non‐genomic signaling mediators MAPK/ERK and PI3K/Akt (Heldring et al. 2024; Menazza and Murphy 2016).

In formulating our hypothesis, we anticipated that a dose‐dependent increase in ERα abundance and phosphorylation of ERK and AKT would occur only in BK‐R and BK‐A. As expected, BK‐A fully matched this expectation, showing a clear dose‐dependent increase in both ERα expression and phosphorylation, consistent with strong activation of both genomic and non‐genomic pathways. BK‐R, however, deviated from this pattern: although in vitro it robustly activated both pathways, at the protein level it exhibited dose‐dependent decreases in ERα and p‐Akt alongside an increase in p‐ERK, a profile more consistent with a feedback regulatory mechanism than with simple inhibition (Rozengurt et al. 2014). This interpretation is supported by the alignment between the p‐ERK increase and the dual pathway activation observed in the cell‐based assays (Bahar et al. 2023) and by the strong physiological readouts (see Figures 4, 5, 6, 7e–h).

Thus, for BK‐R, muted protein shifts reflect negative feedback rather than lack of signaling. In contradistinction to the feedback‐compatible attenuation seen with BK‐R, BK‐E provides no evidence for a feedback response. Transcript‐level changes failed to couple to increases in ERα, p‐ERK, or p‐AKT. Protein effects were not statistically significant. Endpoint data were likewise limited: uterine weight showed no gain (Figure 4d); cortical porosity showed no improvement (Figure 6g); osteocalcin unchanged (Figure 8b). Overall, BK‐E exhibits the weakest engagement and efficacy, indicating modest, tissue‐selective action rather than substantive non‐genomic activation effects (Kocanova et al. 2010; Valley et al. 2008).

To examine whether these mechanistic differences might be related to variations in bioactive phytochemical composition, we performed HPLC analysis. In BK‐R, betanin and isobetanin were identified as prominent constituents, rather than chlorogenic acid (CGA) as seen in BK‐A and BK‐E. This compositional difference is consistent with the observation that BK‐R's strong estrogenic activity does not correlate with CGA concentration, suggesting that betanin‐related compounds may play a primary role in its bioactivity (Chandrasekaran and Joseph 2025; Esatbeyoglu et al. 2015; Mohammed et al. 2025).

BK‐A contained CGA as a major phenolic constituent yet showed estrogenic potency beyond what CGA alone would predict. Among the three extracts, BK‐A consistently produced the strongest effects in vitro and in vivo, implying that additional constituents—likely flavonoids or other phenolics—synergize with CGA to enhance efficacy (Naveed et al. 2018; Nguyen et al. 2024). BK‐E was also CGA‐rich but exhibited the weakest estrogenic responses. Its activity was confined to genomic ER targets with no detectable engagement of non‐genomic pathways at the protein level. Even so, BK‐E conferred modest bone‐protective effects in vivo, consistent with a comparatively less potent, tissue‐selective (SERM‐like) candidate (Enokuchi et al. 2020; Naveed et al. 2018; Nguyen et al. 2024).

Collectively, our results identify BK‐R and BK‐A as full ER agonists and BK‐E as a SERM‐like candidate, with comparatively lower efficacy for BK‐E. BK‐R's estrogenic effects appear to be driven primarily by betanin‐related compounds rather than CGA, while BK‐A, despite containing CGA, demonstrated superior potency likely through the synergistic action of multiple constituents. BK‐E, although limited to genomic ER activation, delivered modest bone‐protective effects with minimal uterotrophic stimulation, underscoring tissue selectivity rather than broad ER agonism. This pattern indicates that CGA alone does not predict estrogen‐like efficacy.

These findings position BK‐A as the most robust candidate for broad menopausal symptom relief, likely due to synergistic action among multiple bioactives including CGA; BK‐R as a potent full ER agonist whose strong reproductive and skeletal efficacy appears driven primarily by betanin‐related compounds. By contrast, BK‐E exhibited the weakest efficacy: despite being CGA‐rich, it failed to meaningfully activate non‐genomic pathways and produced only modest estrogenic effects in vivo. This discrepancy underscores that CGA content alone cannot predict estrogen‐like efficacy. Further studies should clarify which phytochemicals beyond CGA drive robust responses, evaluate clinical relevance, and determine whether observed activities derive from individual molecules or complex phytochemical interactions. Collectively, these results highlight BK‐A and BK‐R as strong candidates for phytoestrogen‐based therapeutics, whereas BK‐E shows limited potential under the tested conditions. Importantly, given the adverse effects associated with conventional hormone replacement therapy (HRT), our findings suggest that BK‐A and BK‐R may represent promising natural alternatives with improved safety margins, offering a potential path toward developing phytoestrogen‐based strategies to alleviate menopausal symptoms.

## 5 Conclusion

This study demonstrates that the natural extracts BK‐R, BK‐A, and BK‐E exhibit distinct estrogen‐like activities with divergent mechanistic and physiological profiles. BK‐A emerged as the most potent and consistent candidate, producing robust benefits across uterine, vaginal, and skeletal endpoints, with evidence of dual activation of genomic and non‐genomic ER pathways. BK‐R also acted as a full ER agonist, showing broad reproductive and skeletal efficacy, with its activity linked to betanin‐related compounds rather than chlorogenic acid (CGA).

In contrast, BK‐E failed to increase uterine weight and showed only minor histological and skeletal improvements that were inconsistent across endpoints. Protein‐level signaling analysis confirmed the absence of significant changes in ERα, pERK, or pAKT protein levels, indicating that BK‐E does not meaningfully engage either genomic or non‐genomic pathways. Although BK‐E conferred modest and selective bone protection, its overall efficacy was weak and limited compared to BK‐R and BK‐A.

Collectively, these findings indicate that CGA content alone does not predict estrogen‐like efficacy. BK‐A likely achieves its potency through synergistic interactions among multiple bioactives, while BK‐R derives its effects primarily from betalain pigments. BK‐E, despite being CGA‐rich, showed minimal biological activity. Importantly, given the adverse effects associated with conventional hormone replacement therapy (HRT), BK‐A and BK‐R may represent promising natural alternatives with improved safety margins, offering a potential path toward the development of phytoestrogen‐based therapeutic strategies for alleviating menopausal symptoms.

## 6 Limitation & Future Work

CGA content alone does not fully explain the observed biological responses. It is necessary to determine CGA's standalone activity and its potential synergy with betanin, isobetanin, and other constituents. It is also necessary to characterize absorption, distribution, and target‐tissue exposure to link dose with effect. Controlled human studies in peri‐/postmenopausal women are needed to evaluate efficacy under real‐world conditions, and longer‐term safety monitoring (approximately 6–12 months), including assessments for interactions with commonly used medicines, is needed to define an acceptable risk profile.

## Author Contributions

**Tae‐baek Lee:** conceptualization (lead), data curation (lead), formal analysis (lead), investigation (lead), writing – original draft (lead). **Eunju Jang:** data curation (supporting), methodology (supporting), validation (supporting). **Soobin Choi:** formal analysis (supporting), investigation (supporting), methodology (supporting). **Lisa Tonini:** validation (supporting), writing – original draft (supporting), writing – review and editing (supporting). **Da‐Ye Nam:** data curation (supporting), formal analysis (supporting). **Jae‐Hoon Kim:** methodology (supporting), supervision (supporting). **Hyuk‐Joon Choi:** funding acquisition (supporting), project administration (lead), resources (lead). **Changhwan Ahn:** conceptualization (supporting), supervision (lead), writing – review and editing (lead), funding acquisition (lead).

## Conflicts of Interest

The authors declare no conflicts of interest.

## Supporting information


**Figure S1:** Quantitative analysis of growth‐arrest morphology.
**Table S1:** Primer list and sequences used in this study.

## Data Availability

The data supporting the findings of this study are available from the corresponding author on reasonable request.
