# Supplementary material for: Improvement of Menopausal Symptoms by Beta vulgaris, Artemisia princeps, and Eleutherococcus senticosus via Estrogen Pathway Activation in MCF‐7 Cells and OVX Mice
Source: Food Sci Nutr. 2025 Nov 14;13(11):e71211. doi: 10.1002/fsn3.71211 (PMC12616504; doi:10.1002/fsn3.71211)
Supplement: Supplementary file 1 — Figure S1: Quantitative analysis of growth‐arrest morphology. Table S1: Primer list and sequences used in this study. [file FSN3-13-e71211-s001.docx]

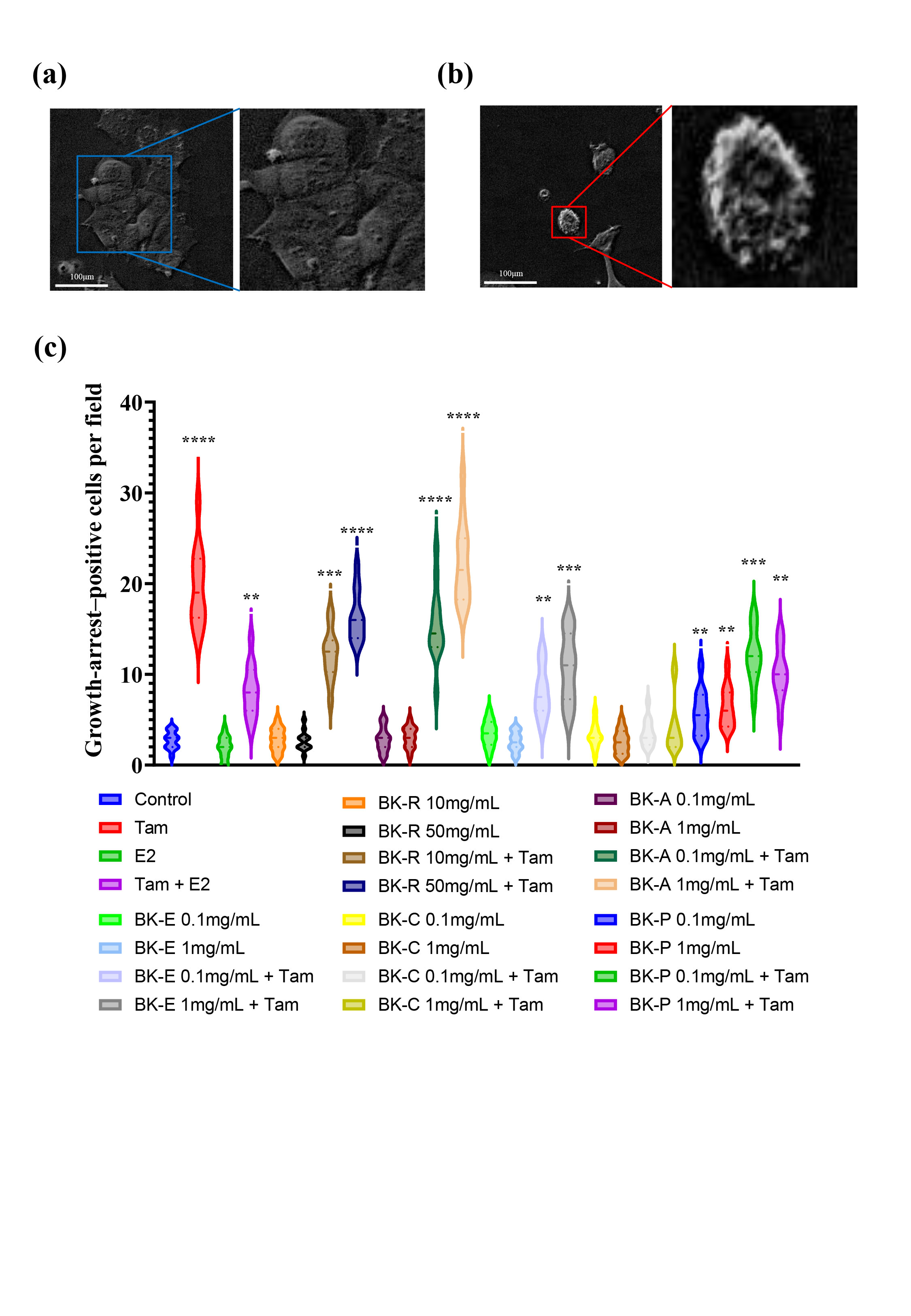


**Supplementary Figure S1. Quantitative analysis of growth-arrest morphology.**

(a) Representative normal cell morphology (blue box); magnified view at right. Scale bar = 100 µm. (b) Representative growth-arrest–positive cell (red box); magnified view at right. Cells appearing bright, highly condensed and shrunken with reduced nucleus-to-cytoplasm ratio required. Scale bar = 100 µm for overview; insets at higher magnification. (c) Distribution of growth-arrest–positive cells per field (cells/field) displayed as violin plots; width reflects data density, individual dots denote single fields (each dot = one field), and the solid line marks the median. Higher values indicate more growth-arrest morphology per field. n = 12 fields per group. Statistics: negative-binomial GLMM (treatment = fixed effect; experiment/plate = random effect) with Benjamini–Hochberg adjustment; comparisons vs. vehicle(control) (***p* < 0.01; ***p < 0.001; *****p* < 1 × 10⁻⁴).

**Supplementary Table S1. Primer List and Sequences Used in This Study**
